# Influence of Wheelchair Type on Kinematic Parameters in Wheelchair Rugby

**DOI:** 10.3389/fspor.2022.861592

**Published:** 2022-06-03

**Authors:** Sadate Bakatchina, Thierry Weissland, Florian Brassart, Ilona Alberca, Opale Vigie, Didier Pradon, Arnaud Faupin

**Affiliations:** ^1^Laboratory Physical Activity Impact on Health (IAPS), University of Toulon, Toulon, France; ^2^Laboratory of Material to System Integration (IMS), University of Bordeaux, Pessac, France; ^3^Pole Parasport - ISPC Synergies, CHU Raymond Poincaré, APHP, Garches, France

**Keywords:** wheelchair rugby, sprint, peak velocity, asymmetry, inertial measurement unit

## Abstract

**Introduction:**

In wheelchair rugby, players use either an offensive or defensive wheelchair depending on their field position and level of impairment. Performance of wheelchair rugby players is related to several parameters, however it is currently unclear if differences in performance are related to wheelchair type or no: the effect of wheelchair type on performance variables has not been evaluated. The aim of this study was to compare offensive and defensive wheelchairs on performance variables during a straight-line sprint.

**Methods:**

Thirteen able-bodied people performed two 20 m sprint trials: one with an offensive and one with a defensive wheelchair. Data were collected using inertial measurement units fixed on the wheelchair. Peak wheelchair velocities and left-right asymmetries in peak wheel velocities were measured during the acceleration and constant peak velocity phases. Sprint time, cycle frequency, and mean and maximum velocity were calculated over the entire sprint.

**Results:**

The peak velocities of the first 2 pushes (acceleration phase) were significantly higher with the defensive than the offensive wheelchair (*p* < 0.04 and *p* < 0.02). Mean and maximum sprint velocity were significantly higher (*p* < 0.03 and *p* < 0.04, respectively) with the defensive wheelchair. Cycle frequency and asymmetry did not differ between wheelchairs.

**Conclusion:**

Performance was higher with the defensive than the offensive wheelchair, suggesting that the frequent finding that the higher performance of offensive as compared to defensive players is not related to the use of an offensive wheelchair.

## Introduction

Wheelchair rugby is a high-performance team sport which was included in the Paralympic program in 2000. Wheelchair rugby players have different types of disabilities (IWRF International wheelchail rugby federation, 2021) that may result from conditions such as spinal cord injury, amputation, polio, cerebral palsy, peripheral neuropathy, or congenital limb deficiency (Gee et al., 2018; Bakatchina et al., 2021a; IWRF International wheelchail rugby federation, 2021). For training and during matches, players are often grouped according to their level of impairment: high point (HP), mid-point (MP) and low point (LP) (IWRF International wheelchail rugby federation, 2021). LP players have a low level of physical ability whereas HP players have a high level of physical ability (IWRF International wheelchail rugby federation, 2021). MP players have intermediate level of physical ability. Studies have classified wheelchair rugby players into two groups: LP and HP (Goosey-Tolfrey et al., 2018) or three groups: LP, MP and HP (Usma-Alvarez et al., 2014; Rhodes et al., 2015a; Haydon et al., 2016, 2018a). Others classified players according to the type of wheelchair used during the game: offensive and defensive players (Bakatchina et al., 2021a). The wheelchairs used during matches have been designed for use by players with different levels of impairment. Offensive wheelchairs (OW) have a front bumper to prevent other wheelchairs from hooking them during the game; defensive wheelchairs (DW) have a bumper that allows them to hook and hold other wheelchairs. OWs are shorter and heavier than DWs (Haydon et al., 2016), consequently OW and DW can be differentiated by the mass distribution. LP players use DW and HP players use OW; MP players can use either type, depending on the coach's strategy.

Comparison of wheelchair rugby players using OW or DW found that those who used an OW achieved higher peak velocities during the acceleration and constant peak velocity phases than those who used a DW (Bakatchina et al., 2021a). However, cycle frequency, which is an indicator of injury risk (Boninger et al., 1999), was higher in players using an OW than those who used a DW (Bakatchina et al., 2021a). According to Boninger et al. (1999), gesture repetition such as cycle frequency during manual wheelchair propulsion would more expose the wheelchair users to risks of injury to their upper limbs. The literature indicated that performance in wheelchair rugby players is related to several parameters such as: players' classification (Sarro et al., 2010; Rhodes et al., 2015a,b; Goosey-Tolfrey et al., 2018), training hours (Furmaniuk et al., 2010; Berzen and Shayke Hutzler, 2012) experience in wheelchair using, gender and age. In addition, the performance during wheelchair manual propulsion is related to the rolling resistances which are the forces that oppose wheelchair displacement causing wheelchair deceleration (Sauret et al., 2009). Thus, wheelchair velocity decreases during wheelchair deceleration (Sauret et al., 2009) impacting player's performance in terms of sprint time during straight-line sprint.

HP players are faster and achieve higher peak power and peak velocity compared to LP players during a 15 s sprint on an instrumented ergometer (Goosey-Tolfrey et al., 2018). However, HP players have higher left-right asymmetry in peak wheel velocity (Goosey-Tolfrey et al., 2018). During matches, HP players achieve higher velocities than LP and MP players (Rhodes et al., 2015a,b) and they spend more time performing high-intensity activities and cover higher distances during the game (Rhodes et al., 2015b). Furthermore, the rate of decrease in velocity between the first and second halves of the match is lower in HP than LP players (Sarro et al., 2010). Wheelchair configuration parameters influence performance, for example camber angle, seat height, seat depth and wheel diameter (Faupin et al., 2004; Mason et al., 2011, 2012, 2013). Larger camber angle is associated with higher power generation (Faupin et al., 2004; Mason et al., 2011) and lower velocities during straight-line wheelchair propulsion (Faupin et al., 2004). Large diameter wheels increased 20 m sprint time and maximum velocity compared to small diameter wheels (Mason et al., 2012).

During a wheelchair rugby game, the ability of players to sprint, pivot, and brake while dribbling or holding the ball are key performance variables. During counter-attacks, players must sprint in a straight line. This important ability can be evaluated using the straight-line sprint test (Gee et al., 2018; Haydon et al., 2018b; Bakatchina et al., 2021a). Performance on the test can be evaluated by measuring kinematic variables such as velocities, accelerations and cycle frequencies (Gee et al., 2018; Bakatchina et al., 2021a). Analysis of these variables during the acceleration and constant peak velocity phases (Haydon et al., 2018b; Bakatchina et al., 2021a) is useful when determining the attributes of a wheelchair. Only Bakatchina and collaborators evaluated peak velocities during the acceleration and constant peak velocity phases of a 20 m straight-line sprint on the field; they found that players using an OW achieved higher peak velocities compared to players using a DW. To our knowledge, no study has investigated the specific influence of wheelchair types (OW or DW) during the acceleration and constant peak velocity phases of a 20 m straight-line sprint on the field in wheelchair rugby. However, it is unclear if the difference in performance was related to the wheelchair type or no. Given that the wheelchair is one of the most important parameters of performance in wheelchair sport (Goosey-Tolfrey, 2010), it is important to analyze the impact of the type of wheelchair on kinematic performance variables. This will serve both to optimize wheelchairs and to guide coaches in their allocation of different wheelchair types to different players. To evaluate the specific effects of OW and DW on performance parameters, the inclusion of able-bodied people is important because they are not yet used to a DW or an OW, so they are unbiased participants.

Consequently, the aim of this study was to compare kinematic variables between OW and DW during the acceleration and constant peak velocity phases of a 20 m straight-line sprint, using IMUs. We hypothesized that: (i) peak velocities during the acceleration and constant peak velocity phases would be higher with the OW, (ii) asymmetry and cycle frequency across the whole sprint would be higher with the OW, exposing the user at risk of injury, and (iii) The rolling resistance would be greater with the DW.

## Materials and Methods

### Participants

A total of 13 able-bodied adults (7 females and 6 males) (Table 1) trained in wheelchair propulsion (see below) were included. None had experienced any upper limb injuries or pain within 6 months preceding the study. All participants were informed of the purpose of the study and any risks that may arise during the test; they all provided informed consent. We chose to perform this study in able-bodied people because we wished to evaluate the specific effects of wheelchair type without the confounding factor of disability; furthermore, studies have shown that trained able-bodied people provide consistent results in experiments using manual wheelchairs (van der Woude et al., 2003; Faupin et al., 2008). The study was approved by the National Ethics Committee for Research in the Physical Activity and Sports Sciences (CERSTAPS N° 2018-16-07-26).

**Table 1:** Individual anthropometric characteristics: gender, age, mass, and height.

	**Gender**	**Age** **(years old)**	**Mass** **(kg)**	**Height** **(cm)**
AB1	M	20	67	173
AB2	M	20	72	179
AB3	M	21	69	175
AB4	F	20	59	163
AB5	F	22	55	162
AB6	F	23	68	165
AB7	F	21	52	163
AB8	M	23	70	185
AB9	F	21	66	172
AB10	M	20	77	176
AB11	F	21	63	170
AB12	F	22	80	181
AB13	F	21	50	164
M (Q1; Q3)	8F; 5M	21 (20; 22)	67 (59; 70)	172 (164; 176)

*M (median), Q1 (first quartile) and Q3 (third quartile)*.

### Wheelchairs

**Figure 1:**
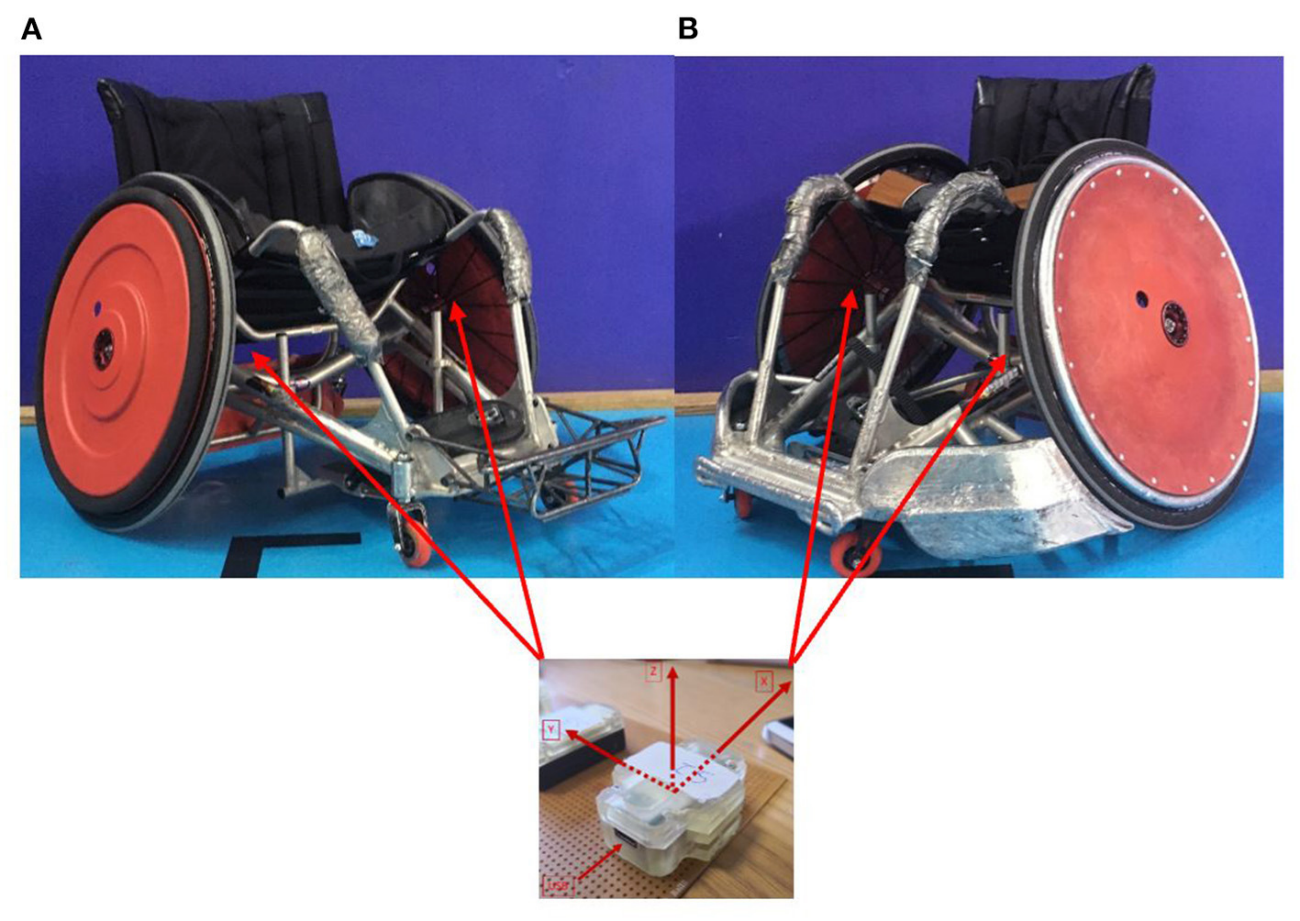
Location of the inertial measurement units on the wheels. **(A)** Defensive wheelchair. **(B)** Offensive wheelchair (2).

According to Haydon et al. (2016), there are two typical wheelchairs: OW & DW. All participants included in current study used one typical DW (Figure 1A) and one typical OW (Figure 1B). The OW weighed 21.8 kg, had a camber angle of 18° and 25-inch wheels (Table 2). The DW weighed 20.7 kg, had an 18° camber and 25-inch wheels (Table 2). We measured frame length, seat length and seat angle (Table 2) according to Haydon et al. (2016). We checked the function of the front casters and rear wheels of each wheelchair before testing.

**Table 2:** Wheelchairs characteristics.

	**Mass** **(kg)**	**Seat** **angle** **(°)**	**Camber** **angle** **(°)**	**Frame** **length** **(m)**	**seat** **length** **(m)**	**Wheel** **diameter** **(inch)**
OW	21.8	22.4	18	0.53	0.38	25
DW	20.7	26.4	18	0.68	0.36	25

*OW, Offensive wheelchair; DW, Defensive wheelchair*.

### Inertial Measurement Unit (IMU)

IMUs are composed of a gyrometer, an accelerometer and a magnetometer which, respectively, allow the measurement of rotational velocity (Usma-Alvarez et al., 2011; van der Slikke et al., 2016; Bakatchina et al., 2021a,b), acceleration (Usma-Alvarez et al., 2011; van der Slikke et al., 2016; Haydon et al., 2018b) and orientation with respect to the magnetic north. We used 2 IMU: 128 Hz, 3 × 3 (accelerometer, gyrometer, magnetometer, and Bluetooth module, WheelPerf System, AtoutNovation, France) (Figure 1) and synchronized them with a tablet computer using Bluetooth version 4.0 technology as described by Bakatchina et al. (2021b).

### Test Protocol

Prior to the test, participants underwent five 2 h training sessions in wheelchair propulsion. We followed the description by Alberca et al. (2021), thus the training included: forward, backward and slalom propulsion over 5, 10, and 20 m. At the beginning of each training session, participants performed a 5–10 min warm-up consisting of forward and backward propulsion and repeated sprints over 20 m. After the warm-up, the participants practiced propelling the wheelchair in a straight line (forward and backward) and around a slalom course at different speeds over 5, 10, and 20 m using both types of wheelchairs. Just before the test, they warmed up for 8–10 min as described by Bakatchina et al. (2021a). They then performed one maximum velocity 20 m sprint with OW and one maximum velocity 20 m sprint with DW, recovery time between both sprints was 10 min. A standing start was used (participants started 20 cm from the starting line). No instructions were given regarding trunk movement during the sprint. Participants sprinted up to the finish line and slowed after crossing the line. The tests were performed in a sports hall on parquet flooring. The same OW and DW were used by all participants and the order of the wheelchairs was randomized.

Rolling resistance tests were then performed with a 20 kg mass placed on the front (first condition) and the rear (second condition) of each wheelchair type seat as described by Bascou et al. (2019). For each condition (forwards and backwards), six trials of deceleration test were performed with each wheelchair type over 5 m. Trials were performed by the experimenter who pushed the wheelchair and stopped it manually on 5 m. Each deceleration test was performed as reported by Bascou and collaborators: “(1) 2 s static phase on a departure mark fixed on the ground, (2) clean manual push to accelerate the manual wheelchair between two 1 m-separated marks, (3) deceleration while verifying the straightness of the manual wheelchair path, (4) clean manual stop between two ending marks, (5) 2 s static phase” (Bascou et al., 2019).

### Data Processing

We placed one IMU on each rear wheel (Figure 1) as described by Bakatchina et al. (2021a). They were positioned between two spokes near the hub and aligned vertically with respect to the horizontal axis of the wheel plane, with the z-axis perpendicular to the vertical axis of the wheel plane. We calculated the rotational velocity of the wheel around the z-axis as described by Fuss (2012) using the gyrometer data. To remove random noise, we used a Butterworth filter (fourth-order zero lag: low-pass-filtered) (Cooper et al., 2002; Bergamini et al., 2015) with a cut-off frequency of 8 Hz (Bakatchina et al., 2021a).

**Figure 2:**
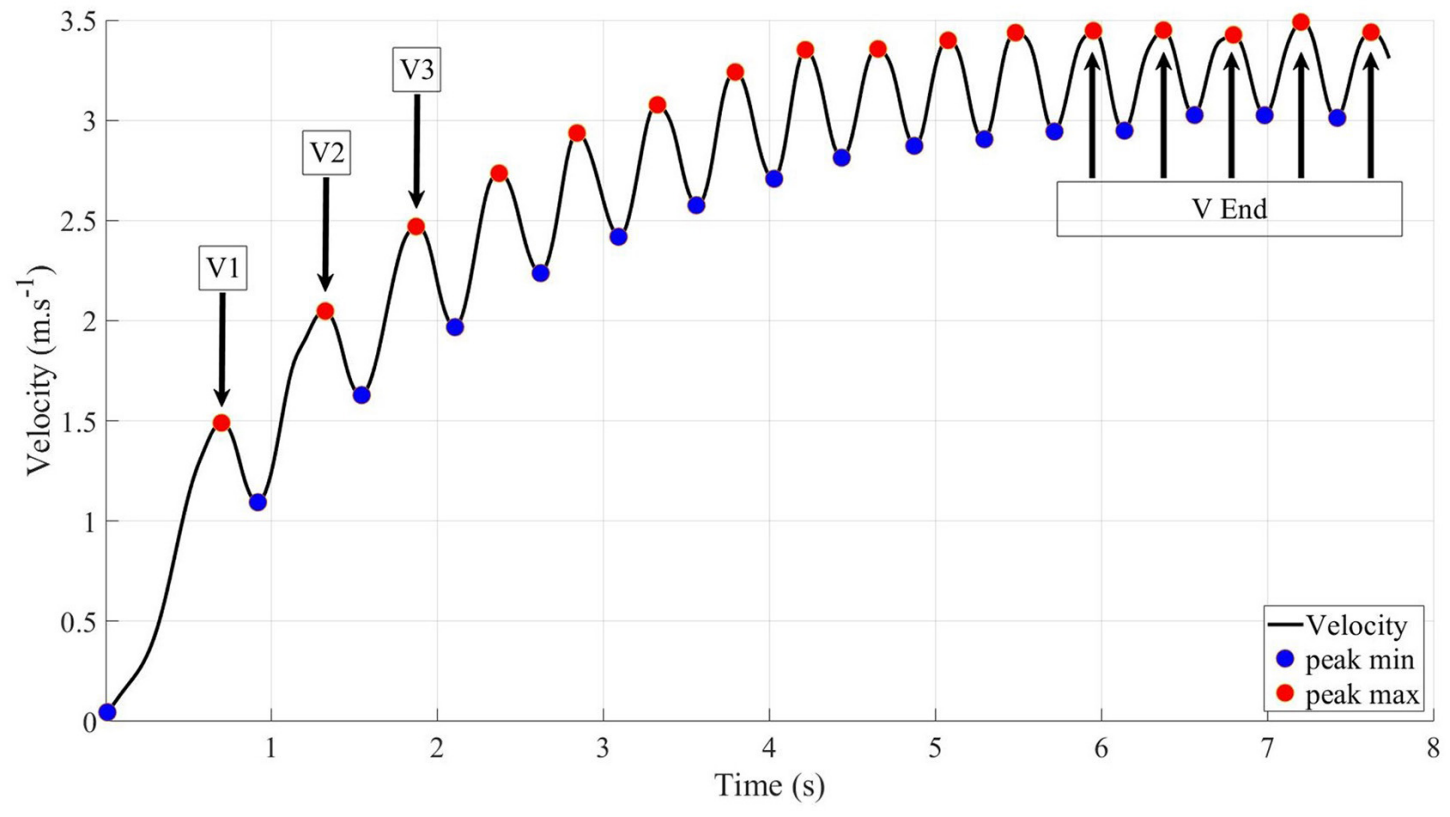
The first three peak velocities (V1, V2, and V3) on the acceleration phase and five peak velocities (V End) on the constant peak velocity phase (2).

We used the finder function of the Matlab program to identify the minimum and maximum peaks on the rotational velocity curve as described by Bakatchina et al. (2021a). Kinematic parameters were calculated during the acceleration and constant peak velocity phases. The acceleration phase was defined as the first 3 pushes and the constant peak velocity phase as the last five pushes (Bakatchina et al., 2021a) (Figure 2).

We calculated the following performance variables according to Bakatchina et al. (2021a): the peak velocity of each of the first 3 pushes (V1, V2, and V3), the mean velocity of the last 5 pushes (Vend) during the constant peak velocity phase, sprint time, and mean and maximum velocity over the whole sprint. Cycle frequency (F) was defined as the number of cycles per minute and asymmetry (Asy) as the difference between the peak velocities of the right and the left wheels (Equation 1) (Goosey-Tolfrey et al., 2018; Bakatchina et al., 2021a).

(1) $$\begin{array}{r} Asy=\frac{\left|\text{V dh}-\text{V non}-\text{dh}\right|}{\text{V non}-\text{dh}}\;\times 100\;\left(\%\right) \end{array}$$

where Asy: asymmetry; V dh: peak velocity of the dominant hand; V non-dh peak velocity of the non-dominant hand (2). Dominant hand was the hand that achieved higher peak velocity and non-dominant hand achieved lower velocity.

**Figure 3:**
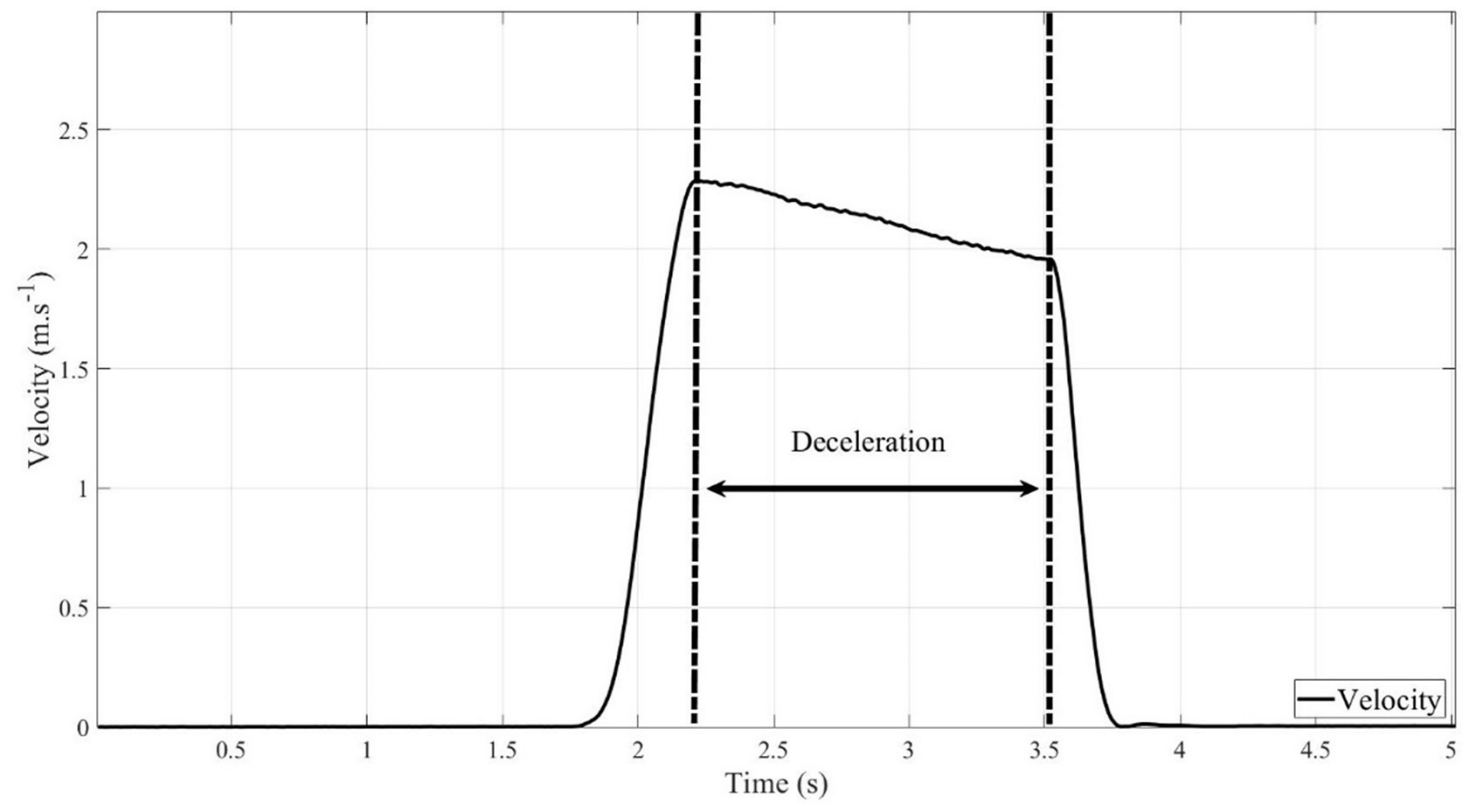
Deceleration on the velocity curve.

During propulsion, drag force (DF) is composed of rolling resistance forces (RRF), air resistance forces (ARF), gravitational forces (GF), and internal frictional forces (IFF) (Equation a) (van der Woude et al., 2001; Rietveld et al., 2021). ARF, GR and IFF are negligible (Equation b) as indicated by Rietveld et al. (2021). We calculated deceleration values (Figure 3) by deriving the linear velocity (c) of the wheels. We then calculated rolling resistance according to Equation (d).

$$\begin{array}{l} \text{DF}=\text{ RRF}+\text{ARF}+\text{GR}+\text{IFF }\left(a\right)\\ \text{DF}=\text{ RRF }=\text{ m}.\text{a }\left(b\right)\\ \text{a}=\frac{\text{d}\left(\text{v}\right)}{\text{t}}\;\left(c\right)\\ \text{RRF }=|\text{m}.\text{a}|\;\left(d\right) \end{array}$$

where m: mass of the wheelchair and the 20 kg additional masses; a: deceleration value; v: linear velocity.

Sprint times were also collected using cell gates (Brower Timing Systems, WITTY.GATE). The cell gates were placed at the start and finish lines connected to an electronic timer allowing to display the time of each sprint after.

### Statistical Analyses

We used velocity data from the (Bakatchina et al., 2021a) and G^*^Power 3.1 software to determine the minimum number of participants required for this study. This minimum number found is 8, which is less than the number of participants included in our study. Because the distribution of the variables was not normal according to the Lilliefors normality test, we calculated medians (M) and first (Q1) and third (Q3) quartiles. We compared variables between the OW and DW using the Wilcoxon test. We calculated effect sizes for all variables: low (*r* < 0.3), medium (0.3 < *r* < 0.5), and large (*r* ≥ 0.5). STATISTICA version 7.1 was used for all statistical analyses and *p* < 0.05 was considered as statistically significant.

## Results

**Figure 4:**
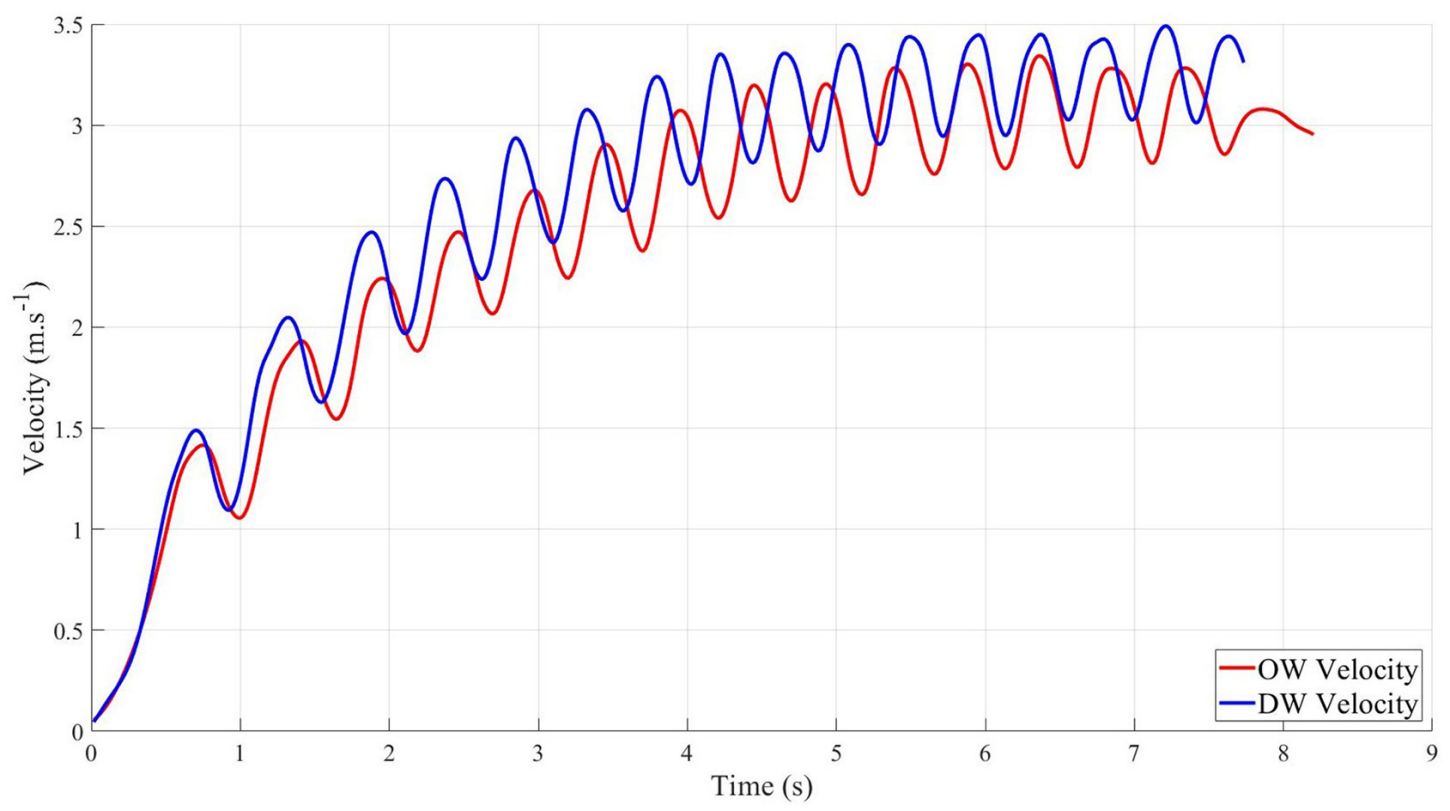
Example of velocity curves of offensive wheelchair (OW) and defensive wheelchair developed by typical participant during 20 m sprint.

Sprint times were faster with DW than OW (Table 3). Significant differences were found in terms of velocities between both wheelchairs (Figure 4). Peak velocity values V1 and V2 were higher with DW (Table 3). Mean (Vav) and maximum velocity were significantly higher with DW (Table 3). The values of V3 and Vend did not differ significantly between wheelchairs (Table 3). The magnitude of these effects was either medium or large (range from 0.44 to 0.88).

**Table 3:** M (median), Q1 (first quartile) and Q3 (third quartile) of velocities (m.s^−1^); time (s); asymmetry (%): relative difference in velocity between the left and right side; F (cycle.min^−1^): Cycle Frequency and RR (N): rolling resistance.

		**OW M** **(Q1; Q3)**	**DW M** **(Q1; Q3)**	***p***	***r***
Velocity	V1	1.83 (1.75; 1.98)	2.00 (1.82; 2.09)	0.04*	0.55
(m.s-1)	V2	2.36 (2.17; 2.57)	2.59 (2.33; 2.69)	0.02*	0.63
	V3	2.72 (2.48; 2.89)	2.94 (2.80; 3.15)	(0.11) NS	0.44
	Vend	3.75 (3.43; 4.04)	3.91 (3.56; 4.21)	(0.07) NS	0.49
	Vmax	3.84 (3.60; 4.30)	4.09 (3.67; 4.37)	0.03*	0.59
	Vmean	2.70 (2.42; 2.82)	2.74 (2.59; 3.06)	0.04*	0.55
Time (s)	T	7.42 (7.12; 8.26)	7.31 (6.57; 7.72)	0.04*	0.56
Asymetries (%)	Asy1	4 (3; 5)	6 (3; 8)	(0.27) NS	0.30
	Asy2	3 (1; 7)	3 (2; 6)	(0.80) NS	0.07
	Asy3	5 (3; 6)	4 (1; 6)	(0.13) NS	0.42
	Asy end	3 (3; 4)	2 (2; 3)	(0.08) NS	0.47
Cycle frequency (cycle.min^−1^)	F	94.74 (85.80; 105.83)	94.32 (84.04; 101.83)	(0.88) NS	0.16
Rolling resistance (*N*)	RR front	6.26 (5.35; 7.05)	9.34 (8.17; 9.74)	0.002**	0.88
	RR rear	5.57 (5.05; 6.27)	7.88 (6.64;8.5)	0.004**	0.81

DW, defensive wheelchair; OW, offensive wheelchair. Significant differences (*p < 0.05, **p < 0.01). No significant difference (NS). Effect size (r).

In addition, neither cycle frequency nor asymmetry during the acceleration and constant peak velocity phases differed between the wheelchairs (Table 3).

Rolling resistance values differed significantly between OW and DW for each condition. For both the first condition (additional mass placed on the front of the seat) and second condition (additional mass placed on the rear of the seat), rolling resistance was significantly higher with DW compared to the OW, with large effect sizes (Table 3).

## Discussion

Performance in wheelchair rugby players is related to several such as: players level of impairment, experience in a wheelchair using, training hours, physical and technical capacities, gender, and age. Currently, it is unclear if differences in performance between rugby wheelchair players could be related to their wheelchair type or no. Consequently, we investigated the influence of an OW and a DW on kinematic variables during a straight-line sprint. To our knowledge, this is the first study to compare the impact of wheelchair type on kinematic variables in wheelchair rugby. Our results showed that all kinematic performance variables were higher for the DW than the OW. In addition, no difference in terms of asymmetry was found between both wheelchairs. However, the results of this study confirmed our third hypothesis that the rolling resistance would be greater with the DW.

Peak velocities during the first 2 pushes (i.e., acceleration phase) were significantly higher with the DW than the OW, with a large effect size (*r* ≥ 0.55). These results are not similar to those of Bakatchina et al. (2021a) who compared players using a DW with players using an OW and found that players using an OW developed higher peak velocities than the players using a DW during the acceleration phase. The mean and maximal velocity during the 20 m sprint were also higher and sprint time was shorter with the DW, with large effect sizes (*r* ≥ 0.55). These differences between our results and those of Bakatchina et al. (2021a) can be attributed by the fact that the participants in our study are able-bodied people while those in Bakatchina et al. (2021a) were people with impairments.

The difference in terms of first and second peak velocities, mean velocity, maximal velocity and sprint time found between the OW and the DW in the current study could be related to maneuverability, stability and steering during wheelchair propulsion. OW was handy allowing the user to prevent other wheelchairs from hooking it in practice. In addition, the OW was shorter than the DW (Haydon et al., 2016), which could cause more maneuverability leading instability with OW. According to Tomlinson (2000) who studied the managing maneuverability and rear stability of adjustable manual wheelchairs, they showed that the stability decreases as maneuverability improves. The instability of OW would cause a high variability in kinetic or kinematic variables between both sides of wheelchair as described by Vegter et al. (2013) and Soltau et al. (2015) who compared simultaneous results of two wheels attached to the different sides of the wheelchair. This kinetic or kinematic variables variability between both sides of the wheelchair would cause a steering movement (Wieczorek and Kukla, 2020) which would prevent OW to run in a straight line (De Groot et al., 2002; Soltau et al., 2015) causing a decrease in the performance variables. Steering movement corrections by OW user during a straight-line sprint would lead to increase energy cost (Vegter et al., 2014; Soltau et al., 2015), and causing a decrease performance in terms of mean velocities and sprint time.

Differences in performance between OW and DW could also be related to the user's position in the manual wheelchair. The performance of manual wheelchair players is also related to the wheelchair user position relative to the main axle position (Brubaker, 1986). Thomas et al. (2018) indicated that reclining the wheelchair seat relative to the horizontal axis increased stability in a wheelchair user. According to Haydon et al. (2016), the seat angle of the DW used by LP players was significantly higher than the seat angle of the OW used by HP players. In the current study the seat angle of the DW was slightly higher compared to the OW (26.4° for DW and 22.4° for OW). Consequently, participants would be more stable with the DW during propulsion causing a better sprint time and a high development of peak velocities.

Asymmetry is considered to be related to both decreased performance and increased risk of injury (Vegter et al., 2013; Gagnon et al., 2016). Comparison of asymmetry during straight-line sprinting is important as this is a component of matches. We found no difference in asymmetry between the OW and the DW during either the acceleration or the constant peak velocity phases. The asymmetry values were similar to those reported by Bakatchina et al. (2021a) in a comparison of players using an OW and players using a DW. Cycle frequency is also a key determinant of propulsion injury risk (Boninger et al., 1999). However, our study showed no significant difference in cycle frequency between OW and DW. This contrasts with the findings of Goosey-Tolfrey et al. (2018) who found a higher cycle frequency in HP players than LP players. Cycle frequency may be related to the level of impairment, which would explain the between-group difference in the (Goosey-Tolfrey et al., 2018) study, and the lack of difference in the present study of able-bodied individuals.

Rolling resistance values differed significantly between both wheelchair types for each condition; they were higher with DW than OW in both conditions (when the 20 kg additional mass was placed in front or rear for both wheelchair types). These higher rolling resistance values with DW could be related to the frame length of DW which was higher compared to the OW (Haydon et al., 2016), resulting in a more distribution of DW mass on the front casters. According to Rémy N de et al. (2003) and Sauret et al. (2013), when the mass distribution of the wheelchair-user system is higher on the front of the wheelchair, rolling resistance during wheelchair propulsion is increased.

## Perspectives

In wheelchair rugby, the choice of the wheelchair type (DW or OW) is related to several factors such as: players' physical capacity or coach's strategies. The current results indicate performance in wheelchair rugby could be related to the wheelchair type. For example, MP players may use either an OW or a DW during the game, therefore some wheelchair rugby clubs have two types of wheelchairs for each MP so that the coach can change the role of the MP between seasons or at half-time. Consequently, coaches and MP players could optimize the choice of wheelchair type improving players performance and coach's strategy. Performance is also partly related to the functional capacity of the abdominal muscles (Vanlandewijck et al., 2010); we believe it would be pertinent to review the configuration of the OW to decrease steering movement in HP players during straight-line sprint. In addition, the ability to accelerate and pivot whilst maintaining control of the ball are also key performance variables in wheelchair rugby. Consequently, future studies should compare the performance of these wheelchairs during pivoting tasks such as the 8 test.

## Conclusions

Wheelchair configuration is considered as a key performance variable in wheelchair rugby; few studies have evaluated interactions between the user and the wheelchair. The results of our study suggest that wheelchair type influences performance in wheelchair rugby. Mean and maximal velocity and peak velocity during the acceleration phase were higher with the DW than the OW. Sprint time was also faster with the DW. Cycle frequency and asymmetry, which are risk parameters for injury and indicators of high-performance parameters in HP players, do not appear to be influenced by wheelchair type. These results should provide guidance to coaches in the choice of wheelchair type for MP players.

## Data Availability Statement

The raw data presented in the study are not readily available because the participants did not consent to sharing their data when they entered the study. To access the raw data, please contact us at: sadate.bakatchina@univ-tln.fr.

## Ethics Statement

The studies involving human participants were reviewed and approved by National Ethics Committee for Research in the Physical Activity and Sports Sciences (CERSTAPS No 2018-16-07-26). The patients/participants provided their written informed consent to participate in this study. Written informed consent was obtained from the individual(s) for the publication of any potentially identifiable images or data included in this article.

## Author Contributions

SB contributed to conception, design, organized the database and of the study, performed the statistical analysis and wrote the first draft of the manuscript. TW, FB, IA, OV, DP, and AF wrote sections of the manuscript. All authors contributed to manuscript revision, read, and approved the submitted version.

## Conflict of Interest

The authors declare that the research was conducted in the absence of any commercial or financial relationships that could be construed as a potential conflict of interest.

## Publisher's Note

All claims expressed in this article are solely those of the authors and do not necessarily represent those of their affiliated organizations, or those of the publisher, the editors and the reviewers. Any product that may be evaluated in this article, or claim that may be made by its manufacturer, is not guaranteed or endorsed by the publisher.
